# Supplementary material for: Understanding natural selection in Holocene Europe using multi-locus genotype identity scans
Source: bioRxiv. 2023 Apr 25:2023.04.24.538113. Preprint. [Version 1] doi: 10.1101/2023.04.24.538113 (PMC10168228; doi:10.1101/2023.04.24.538113)
Supplement: Supplement 2 [file NIHPP2023.04.24.538113v1-supplement-2.pdf]

bioRxiv preprint doi: https://doi.org/10.1101/2023.04.24.538113; this version posted April 25, 2023. The copyright holder for this preprint (which was not certified by peer review) is the author/funder, who has granted bioRxiv a license to display the preprint in perpetuity. It is made available under aCC-BY 4.0 International license.

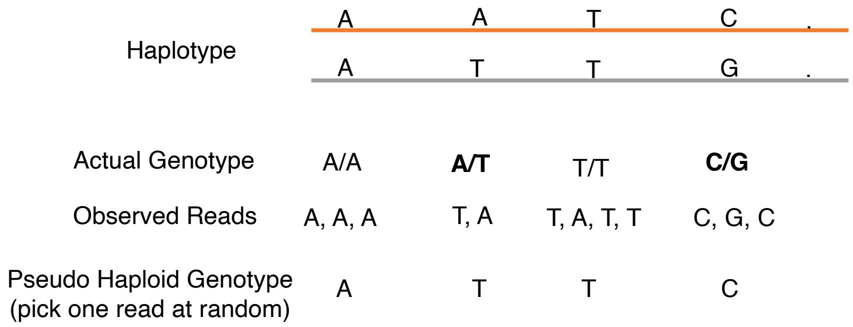

Supplementary Fig. 1: Pseudo haploidization scheme showing random allele calling for the generation of multi-locus genotypes.

Parameter	Modern Samples	Ancient Samples
Mean Missingness (Preprocessing)	0.0131	0.54827
Mean Missingness (Post-processing)	0.53529	0.54827

Supplementary Table 1: Differences between the mean fraction of missing individuals per SNP in modern samples vs. the ancient samples, pre, and post-data processing.

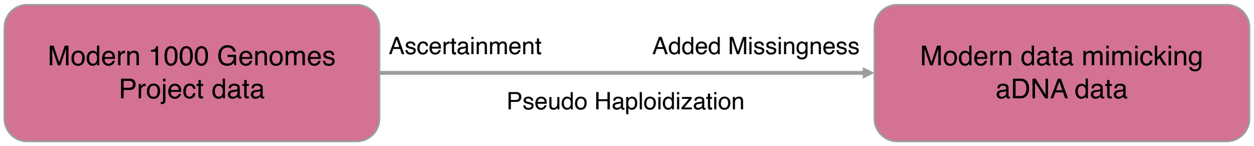

Supplementary Fig. 2: Data processing scheme, we take modern genomic data and apply ascertainment, pseudo-haploidization and add missingness to the data to make it mimic the artefacts of aDNA data used in this study.

bioRxiv preprint doi: https://doi.org/10.1101/2023.04.24.538113; this version posted April 25, 2023. The copyright holder for this preprint (which was not certified by peer review) is the author/funder, who has granted bioRxiv a license to display the preprint in perpetuity. It is made available under aCC-BY 4.0 International license.

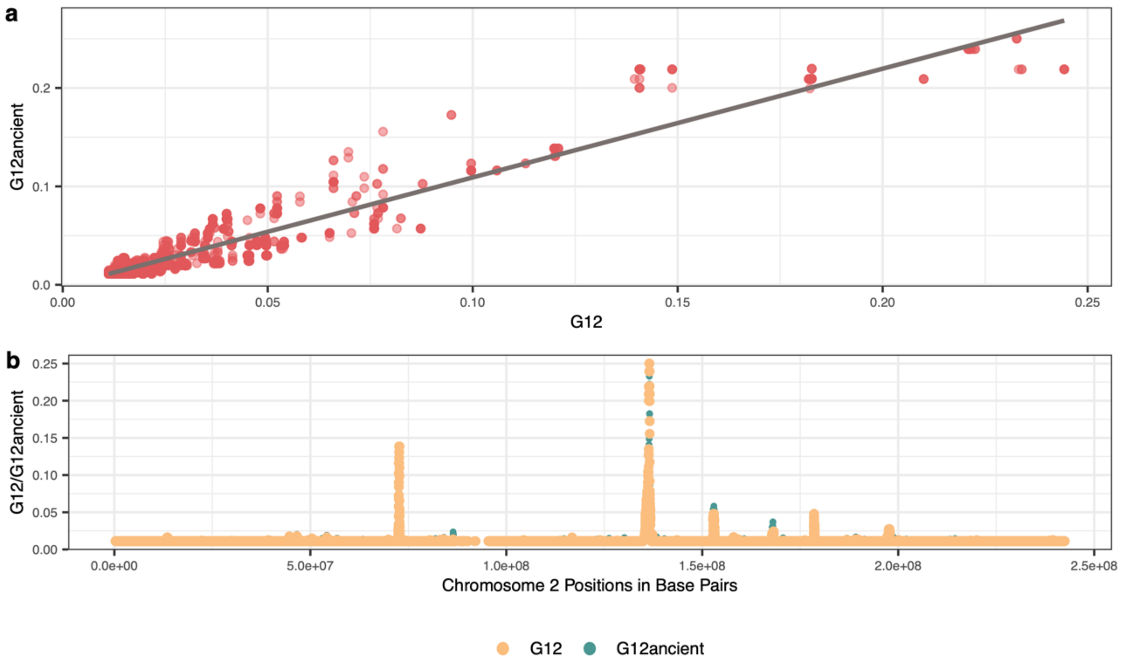

Supplementary Fig. 3*:* Plots showing strong positive correlation between *G12* and $G12_{ancient}$ values for GBR individuals. **a** Scatter plot between *G12* and $G12_{ancient}$ values with a line of best fit showing values are highly correlated. **b** Scatter plot between SNP positions and *G12* / $G12_{ancient}$ values showing that both plots overlay each other to a very higher degree.

bioRxiv preprint doi: https://doi.org/10.1101/2023.04.24.538113; this version posted April 25, 2023. The copyright holder for this preprint (which was not certified by peer review) is the author/funder, who has granted bioRxiv a license to display the preprint in perpetuity. It is made available under aCC-BY 4.0 International license.

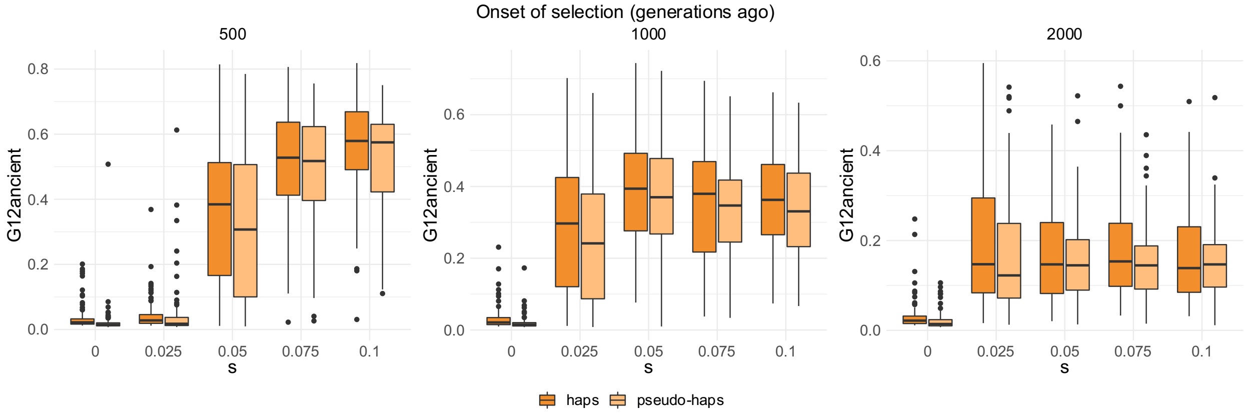

Supplementary Fig. 4: *G12* and $G12_{ancient}$ values for 177 individuals sampled 40 generations ago. No missing data was added to the simulated data. We ran a total of 100 hard sweep simulations for each combination of parameters with mutation rate μ= 1.25×10^{-8} /bp, chromosome length L=5×10^{5} and recombination r = 5×10^{-9} events/bp.

bioRxiv preprint doi: https://doi.org/10.1101/2023.04.24.538113; this version posted April 25, 2023. The copyright holder for this preprint (which was not certified by peer review) is the author/funder, who has granted bioRxiv a license to display the preprint in perpetuity. It is made available under aCC-BY 4.0 International license.

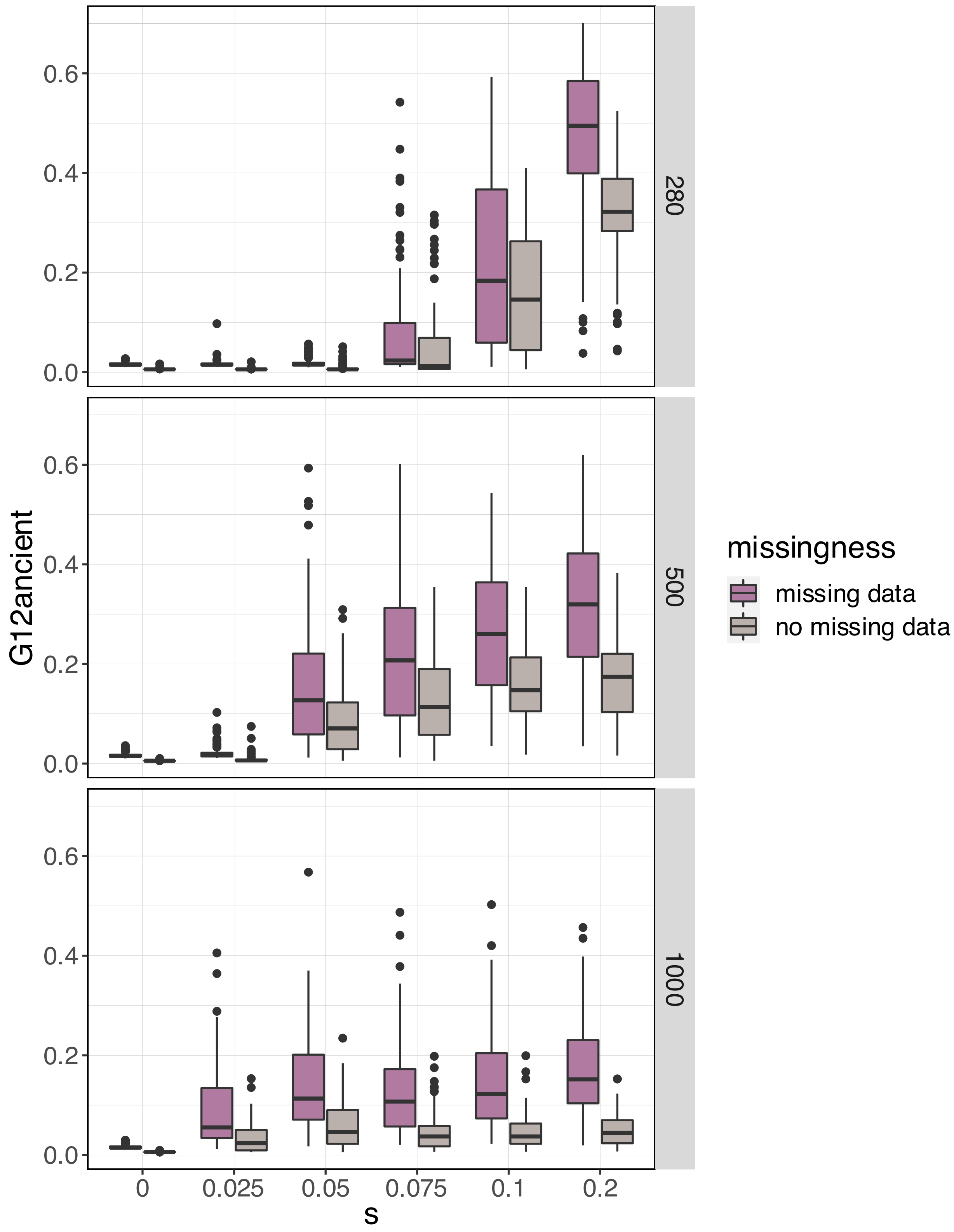

Supplementary Fig. 5: $G12_{ancient}$ values for pseudo-haploidized simulated data from 177 individuals sampled 40 generations ago for a hard sweep model with mean rate of 0.55 missingness per SNP and a standard deviation of 0.23.

bioRxiv preprint doi: https://doi.org/10.1101/2023.04.24.538113; this version posted April 25, 2023. The copyright holder for this preprint (which was not certified by peer review) is the author/funder, who has granted bioRxiv a license to display the preprint in perpetuity. It is made available under aCC-BY 4.0 International license.

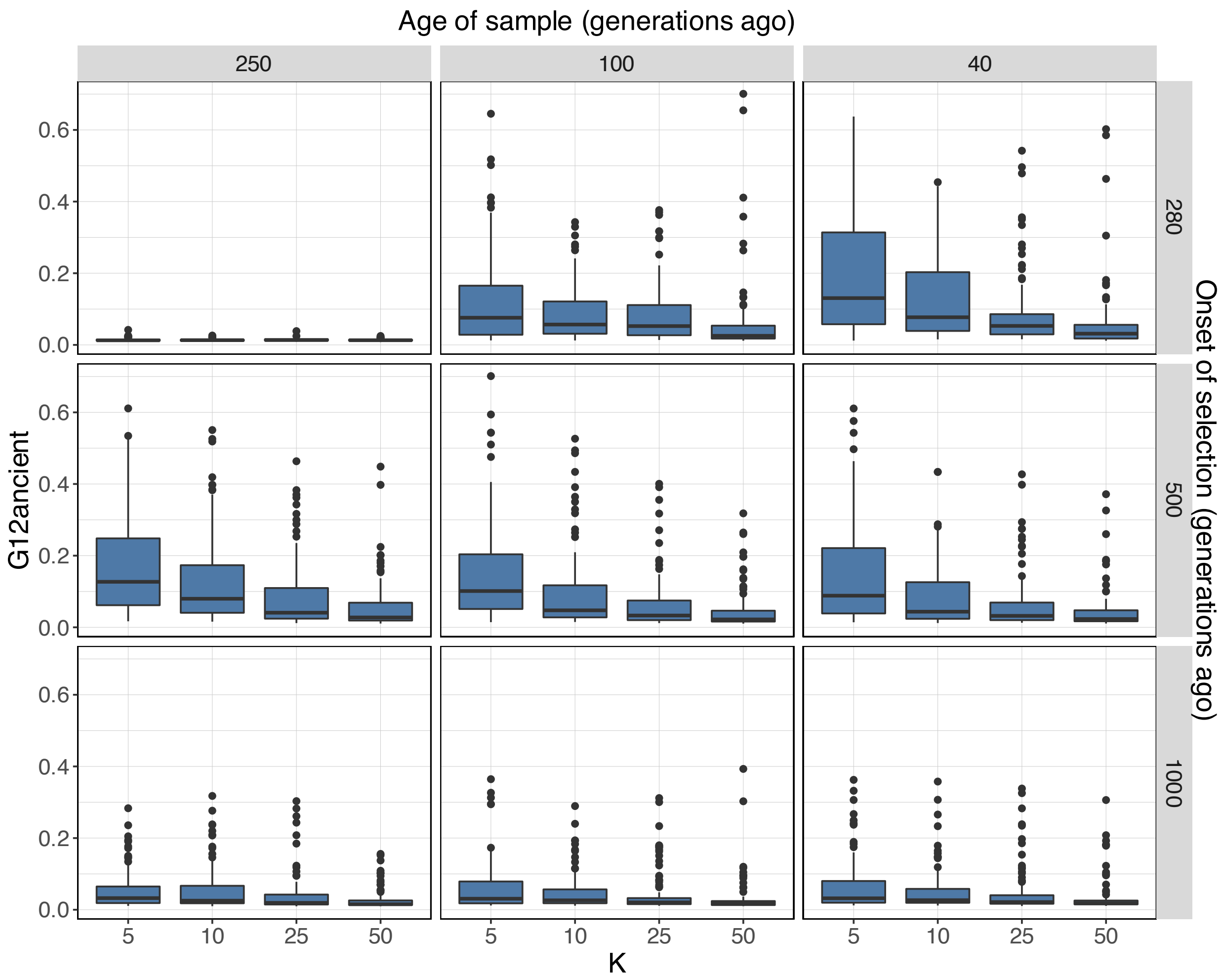

Supplementary Fig. 6: $G12_{ancient}$ values in a soft sweep model. We introduced K beneficial mutations at the time of the onset of selection (rows), for K=*5,10,25* and *50,* where the higher K the softer the sweep. We sampled the population at 3 different time points (columns). We ran a total of 100 simulations for each combination of parameters with mutation rate $\mu = 1.25\times10^{-8}$ /bp, chromosome length $L = 5\times10^{5}$, recombination $r = 5\times10^{-9}$ events/bp and $s = 0.1$. $K = 0$ corresponds to the scenario with no selection.

bioRxiv preprint doi: https://doi.org/10.1101/2023.04.24.538113; this version posted April 25, 2023. The copyright holder for this preprint (which was not certified by peer review) is the author/funder, who has granted bioRxiv a license to display the preprint in perpetuity. It is made available under aCC-BY 4.0 International license.

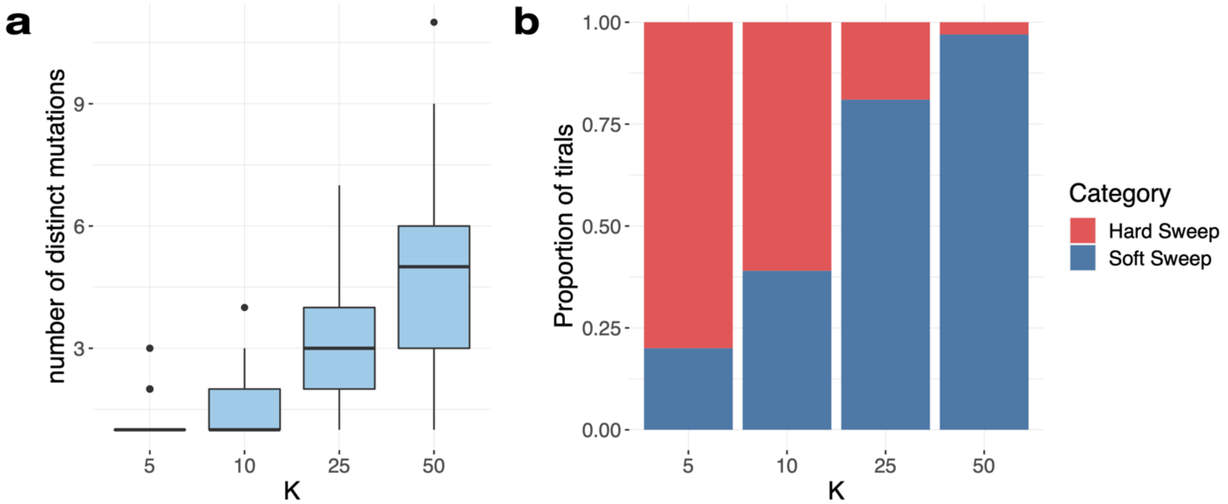

Supplementary Fig. 7: Softness of sweeps starting with *K* distinct mutations introduced 500 generations ago and sampled 40 generations ago. **a** Number of distinct mutations at the time of sampling. **b** Proportion of hard and soft sweeps as a function of *K*.

Gene	Population	Chr	Position	Function
SLC24A5	CEU	15	Band: 15q21.1<br>Start: 48,120,990 bp<br>End: 48,142,672 bp	This locus is one of the major factors influencing skin pigmentation in humans
LCT/MCM6	CEU	2	Band 2q21.3<br>Start 135,839,626 bp<br>End 135,876,443 bp	This enzyme helps to digest lactose, a sugar found in milk and other dairy products
TLR1	CEU	4	Band 4p14<br>Start 38,790,677 bp<br>End 38,856,817 bp	Toll-like receptors are a class of proteins that play a key role in the innate immune system

Supplementary Table 2: The variants of interest that are shown to be under selection by multiple natural selection studies on European genomes.

bioRxiv preprint doi: https://doi.org/10.1101/2023.04.24.538113; this version posted April 25, 2023. The copyright holder for this preprint (which was not certified by peer review) is the author/funder, who has granted bioRxiv a license to display the preprint in perpetuity. It is made available under aCC-BY 4.0 International license.

Epoch	ND across 200 SNP window	Total number of sites	Segregating sites	S/BP	Mean Window Length (bp)
N	0.00002752173	1233013	930906	0.754984	454774 (± 386740)
BA	0.00002817481	1233013	953594	0.773385	454507 (± 386870)
IA	0.00004058848	1233013	962723	0.780789	455005 (± 386849)
H	0.00004152787	1233013	943001	0.764794	454605 (± 386845)

Supplementary Table 3: A table showing the nucleotide diversity calculated for each epoch on a 200 SNP window. We used the vcftools --window-pi option which measures the nucleotide diversity in windows, with the number provided as the window size. We also show the number of segregating sites per base pair.

bioRxiv preprint doi: https://doi.org/10.1101/2023.04.24.538113; this version posted April 25, 2023. The copyright holder for this preprint (which was not certified by peer review) is the author/funder, who has granted bioRxiv a license to display the preprint in perpetuity. It is made available under aCC-BY 4.0 International license.

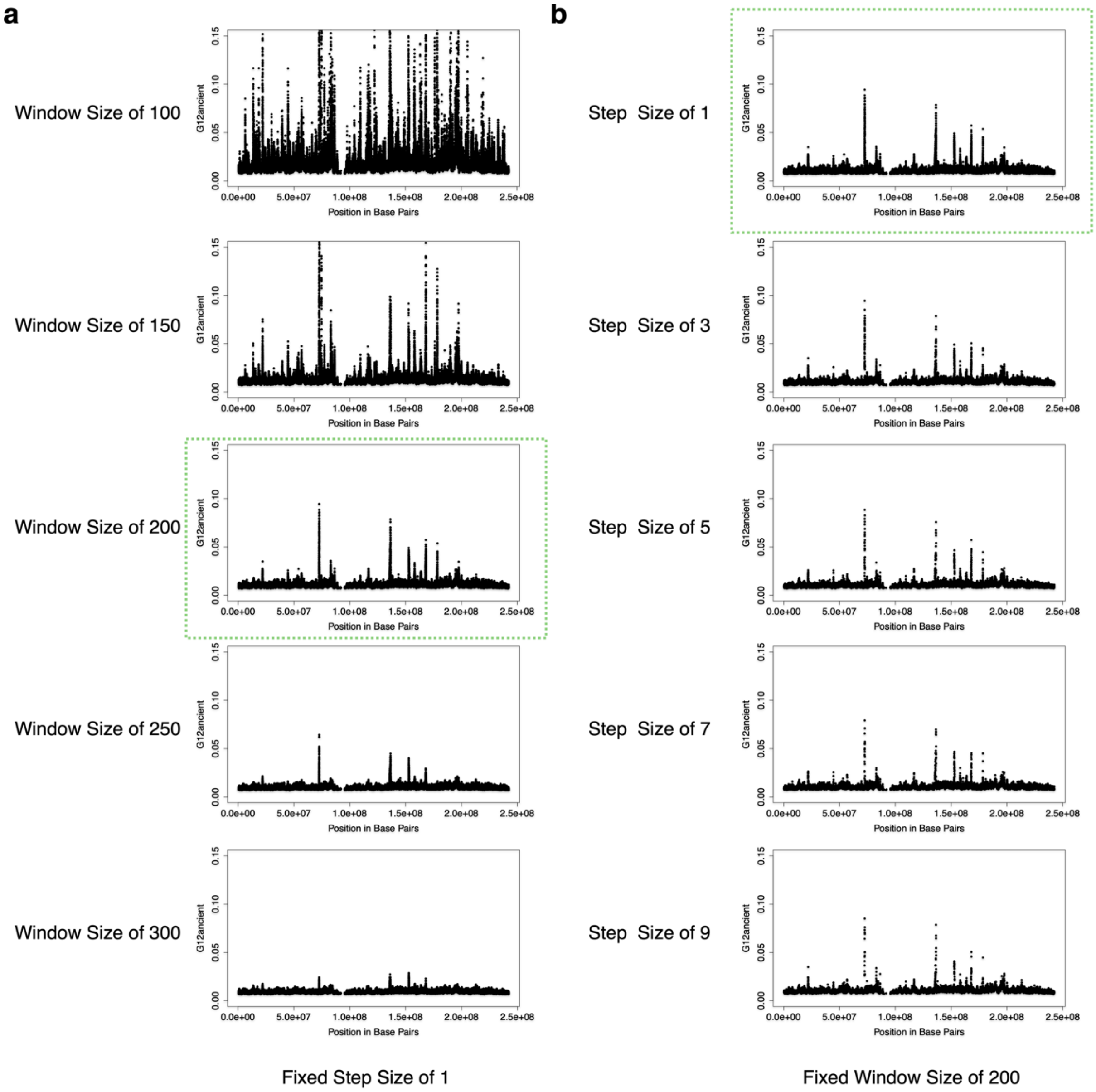

Supplementary Fig. 8: The performance of $G12_{ancient}$ selection scans on different window size and step size values. **a** Variation of window size parameter while keeping step size fixed at 1, we observe window size of 200 as smaller window size resulted in inflated $G12_{ancient}$ values and larger window size resulted in smaller $G12_{ancient}$ values. **b** Variation of step size while keeping window size of 200, we observe that as we increase the step size, we lose a greater number of SNPs considered for calculation $G12_{ancient}$ statistic and it results in loss of SNP density, so we fixed the step size as 1.

bioRxiv preprint doi: https://doi.org/10.1101/2023.04.24.538113; this version posted April 25, 2023. The copyright holder for this preprint (which was not certified by peer review) is the author/funder, who has granted bioRxiv a license to display the preprint in perpetuity. It is made available under aCC-BY 4.0 International license.

**Variable**	***$G12_{ancient}$***	
	R^2 Score	Correlation Coefficient
Window Size	0.013	0.1030
Recombination Rate	0.001	-0.0261
Missingness	0.027	0.1634

Supplementary Table 4: Relationship between parameter choice and $G12_{ancient}$ value suggests that overall $G12_{ancient}$ statistics are unaffected by our choice of parameters.
